# Building train carriages for ciliary transport: (IFT-)A complex task

**DOI:** 10.1038/s42003-023-04426-9

**Published:** 2023-01-23

**Authors:** Francisco Gonçalves-Santos, Maria J. G. De-Castro, Ana R. G. De-Castro, Tiago J. Dantas

**Affiliations:** 1 i3S - Instituto de Investigação e Inovação em Saúde, Universidade do Porto, Porto, Portugal; 2 IBMC - Instituto de Biologia Molecular e Celular, Universidade do Porto, Porto, Portugal

## Abstract

Cilia assembly and function require intraflagellar transport (IFT), a mechanism that uses “trains” to transport cargoes into and out of cilia. While much has been learned about IFT in the past decades, IFT train assembly, loading of cargo and transport regulation have remained poorly understood. In a recent study, Hesketh, Mukhopadhyay and colleagues obtained the complete structure of the IFT-A complex, a key element of IFT trains. By modelling IFT-A into anterograde trains and performing structure-guided mutagenesis, the authors uncover how the IFT-A complex polymerizes and forms carriages to accomplish its distinctive functions.

Cilia are microtubule-based organelles protruding from the surface of most eukaryotic cells. Their unique functional properties allow them to partake in various sensory functions, signalling pathways, fluid flow and cell motility. Hence, it is not surprising that defects in cilia often lead to a wide range of human disorders, collectively called ciliopathies, affecting multiple organ systems and tissues.

Ciliogenesis and ciliary functions rely on a bidirectional transport system, known as intraflagellar transport (IFT; reviewed in ref. ^1^). During anterograde IFT, large molecular machines, called IFT trains, are powered by Kinesin-2 motors to transport cargoes and signalling molecules towards the ciliary tip. Once at the tip, cargo is unloaded, trains are remodelled, and Dynein-2 motors are activated to drive retrograde IFT towards the ciliary base.

Two of the core components of IFT trains are the IFT-A and IFT-B complexes, composed of 6 and 16 subunits, respectively^1^. The IFT-B complex is critical for initiating anterograde IFT as it serves as a scaffold onto which the IFT train is assembled^2^. Conversely, mutants of the IFT-A complex often lead to the development of short bulgy cilia, with strong accumulations of IFT components at their tips, indicating that IFT-A is important for retrograde IFT. Intriguingly, the IFT-A complex has also been shown to play a crucial role in membrane protein import into cilia (reviewed in ref. ^1^). Whether these apparently distinct functions of IFT-A relate to each other remains unclear. Furthermore, while the overall organization of the IFT-A complex has been studied to a certain extent, its assembly and mode of operation in the IFT train remain mostly elusive.

In an elegant and thorough study, Hesketh, Mukhopadhyay and colleagues^3^ reconstituted the human IFT-A complex in vitro and obtained its cryo-electron microscopy (cryo-EM) structure with a resolution ranging from 3.5 to 15 Å. As expected from prior biochemical studies^1^, the authors show that the structure of the IFT-A complex can be subdivided into two modules (Fig. 1). They found that the module priorly known as IFT-A core is actually composed of IFT144, IFT140 and the C-terminal half of IFT122. Intriguingly, this module turns out to be the most flexible region of the IFT-A complex. Conversely, the non-core IFT-A subunits, IFT121, IFT139 and IFT43, together with the N-terminal half of IFT122, actually constitute the most rigid module of this protein complex. With this new structural organization in mind, the authors renamed these modules as IFT-A1 and IFT-A2, respectively^3^.

**Fig. 1:**
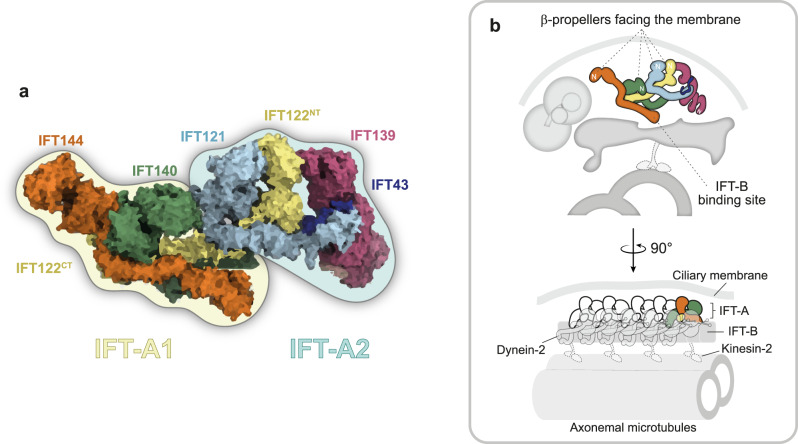
Structural representation of the complete IFT-A complex when docked into the cryo-ET map of anterograde IFT trains from *C. reinhardtii*. **a** The IFT-A complex subdivided into two modules: IFT-A1 (highlighted in light yellow) and IFT-A2 (highlighted in light blue). IFT-A1 encompasses IFT144, IFT140 and the C-terminal region (CT) of IFT122, while IFT-A2 is composed of IFT121, IFT139, IFT43, and the N-terminus (NT) of IFT122. **b** Illustration of IFT-A in orthogonal views relative to IFT-B, axoneme, ciliary membrane, Dynein-2 and Kinesin-2 (inferred position, for context; not to scale). The N-terminal region of each IFT-A subunit is indicated by N in white. Artwork by: Maria J. G. De-Castro.

Zooming into the interactions established within the IFT-A modules, the authors found that the assembly of the IFT-A complex is primarily driven by the dimerization of two sets of subunits: IFT144 with IFT140, and IFT121 with IFT122. Strikingly, both interactions are mediated by the TPR regions in these subunits, all containing a signature α-helix between the second and third TPR. In agreement with the importance of this distinctive domain in IFT-A assembly, the authors found that mutating key interface residues in the IFT140 TPR almost completely disrupts its binding to IFT144. Moreover, expressing this IFT140 mutant in IMCD3 cells fails to rescue the cilium assembly defects associated with the loss of IFT140, underlining the functional relevance of this interaction^3^.

Next, to gain insights regarding IFT-A polymerization, the authors docked their atomic model of the IFT-A complex into the cryo-electron tomography (cryo-ET) map of anterograde IFT trains from *Chlamydomonas reinhardtii* (EMDB: 4304), previously obtained by the Pigino lab^4^. After the more rigid IFT-A2 module was fitted unambiguously into the sub-tomogram average of IFT-A, the more malleable IFT-A1 module was nicely matched to the remaining densities using molecular dynamics flexible fitting. Notably, IFT122 plays a pivotal role in this assembly as it serves as the flexible bridge that connects the two IFT-A modules, facilitating the conformational changes required for IFT-A polymerization into IFT trains^3^ (Fig. 1).

This allocation of IFT-A subunits into the cryo-ET map allowed the authors to also investigate the links established between the IFT-A and IFT-B complexes in anterograde trains. A previous study had already identified an interaction between IFT144 and the IFT-B subunit IFT88^5^. Consistent with this, the authors observed that the C-terminal region of IFT144 positions itself in close contact with the IFT-B complex (Fig. 1). Furthermore, using AlphaFold, the authors successfully mapped the interacting motif of IFT144 with IFT88, and found that this interaction is facilitated by a flexible linker in the IFT88 C-terminus. Mutating conserved residues in the IFT88-binding motif of IFT144, strongly reduced IFT144 loading into cilia, presumably leaving the complete IFT-A complex stranded at the ciliary base. The importance of the link established between these IFT-A and IFT-B components is further underscored by the fact that this IFT144 mutant form was unable to restore cilium elongation in IFT144 knockout cells^3^.

Subsequently, the authors lined up multiple copies of the IFT-A complex into the cryo-ET map of anterograde trains in order to create a molecular model of the polymer from a more biological standpoint. By assigning the different domains of the IFT-A subunits to the various densities in the polymer, they observed that the β-propellers of IFT144, IFT140, IFT122 and IFT121 all face towards the ciliary membrane (Fig. 1), compatible with a role in membrane protein import into cilia. Interestingly, adjacent IFT-A complexes establish multiple connection points between them, both at the level of the β-propellers and of the TPR domains. This interlinked arrangement leads to the formation of empty compartments near the ciliary membrane, which the authors dubbed IFT “carriages”^3^.

Next, the authors show how IFT-A carriages associate with TULP adaptor proteins, the key mediators of membrane protein import into cilia. Using AlphaFold, they identified a channel within the IFT122 CT as the main site for binding TULP proteins. Indeed, mutating key interface residues in this region of IFT122 abolishes the IFT-A/TULP interaction without disrupting IFT-A complex integrity. Importantly, expressing this IFT122^TULPmut^ fails to rescue membrane protein incorporation into cilia in IFT122 knockout cells^3^, similarly to what had been observed in the past upon loss of TULP adaptors^1^.

Upon further examination, the authors also uncovered that key elements of the IFT machinery, namely IFT-A, IFT-B and the retrograde Dynein-2 motor, accumulated at the tips of cilia expressing the IFT122^TULPmut^. These findings suggest that the presence of the IFT-A complex alone is not sufficient to activate retrograde IFT and retrieve these components to the base of cilia. Strikingly, they then found that ICK, a ciliary kinase previously implicated in modulating IFT^6^, was absent from IFT122^TULPmut^ cilia^3^. This exciting result revealed that IFT-A controls the ciliary localization of ICK, allowing the authors to draw a connection between the roles of IFT-A in protein import and retrograde IFT^3^.

In summary, this pivotal study by the Roberts’ lab reshapes our view of the IFT-A structure, while also providing fundamental insights into how IFT-A interacts with IFT-B and polymerizes into IFT-A carriages. Furthermore, it lays out the structural basis for the IFT-A association with TULP adaptors and uncovers its control over the localization of ICK^3^. Finally, this study improves our current understanding of how mutations in ICK, Dynein-2 and IFT-A components lead to severe ciliopathies with overlapping features.

We note that the structure of the IFT-A complex has also been recently worked out in other ciliated organisms^7–9^. Although not highlighted here, these studies provide additional important insights into the assembly and mode of operation of this intriguing complex.
